# Remdesivir-Induced Extreme Sinus Bradycardia in COVID-19

**DOI:** 10.7759/cureus.27307

**Published:** 2022-07-26

**Authors:** Natale Wasef, Steven Hamilton, Tehreem Fatima, Eric Osgood

**Affiliations:** 1 Internal Medicine, Jersey Shore University Medical Center/Saint Francis Medical Center Program, Trenton, USA

**Keywords:** covid-19 infection, cardiotoxicity, reversible bradycardia, drug-induced bradycardia, remdesivir

## Abstract

During the COVID-19 pandemic, there was an urgent need for any medication to help reduce the high death rate experienced during this deadly surge. Remdesivir is an FDA-approved drug for COVID-19 treatment, given its anti-inflammatory properties. Upon extensive literature search, we found two studies and four cases of COVID-19-induced pneumonia treated with remdesivir who were developing bradycardia. In most of these cases, the bradycardia resolved within one-to-two days of holding remdesivir, which correlated with the half-life of remdesivir. Remdesivir was shown to have benefits in COVID-19-induced pneumonia during the COVID-19 surge; however, its use has been controversial. According to the studies, the sinus bradycardia following remdesivir administration does not impact patients’ prognosis in terms of ICU admission and in-hospital mortality. There are multiple case reports noted to report several remdesivir-induced cardiac side effects. In our case, prolonged use and high dosages may induce cardiotoxicity, manifesting as severe bradycardia. Several possible mechanisms for cardiac adverse effects with remdesivir need further investigation and research as COVID-19 remains an active global issue. We present a 53-year-old man hospitalized with COVID-19-induced pneumonia who experienced extreme sinus bradycardia that is likely attributable to remdesivir.

## Introduction

The COVID-19 surge had a high incidence and death rate, with no antiviral shown to be efficacious for its treatment until remdesivir. Remdesivir is an FDA-approved drug for the treatment of COVID-19, which works by inhibiting the RNA-dependent RNA polymerase (RdRp) of coronaviruses, including SARS-CoV-2. Several clinical trials have shown that treatment of COVID-19 pneumonia using remdesivir therapy has faster recovery when compared to placebo [1].

Overall, remdesivir has a favorable adverse effect profile. The most commonly reported adverse events were increased liver aminotransferases, hypersensitivity reactions, nausea, and hypokalemia. Infusion-related reactions have also been reported where patients may experience angioedema, bradycardia, hypotension, and hypoxia [1].

In this case report, we describe a case of remdesivir-induced bradycardia, which is a rare and infrequent side effect reported in the literature.

This article was previously presented as a poster presentation abstract at the American College of Cardiology, Washington, DC, USA, on April 2, 2022. The abstract was published in the Journal of American College of Cardiology in March 2022.

## Case presentation

**Figure 1:**
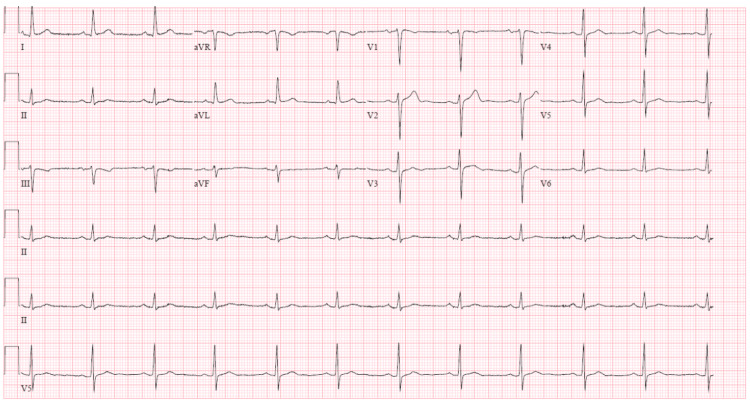
EKG on admission showing normal sinus rhythm with a heart rate of 68 bpm. bpm: Beats per minute.

A 53-year-old man with a known past medical history of diabetes mellitus type 2 presented to our institution complaining of malaise, fatigue, and subjective fever ongoing for four days with concurrent productive cough of yellow sputum for seven days that was progressively worsening. He was unvaccinated for COVID-19 and was recently in contact with a family member who tested positive for COVID-19. On admission, heart rate was 66 beats per minute (bpm), the temperature was 100.6 ºF, respiratory rate was 30 breaths/min, oxygen saturation was 88% on ambient air (improved to 94% on 4 LPM via nasal cannula), and blood pressure (BP) was 139/73 mmHg. Physical exam revealed a regular pulse, S1 and S2 without rubs, murmurs, or gallops. Breath sounds were equal, bronchial, and reduced bilaterally. Coarse rales were appreciated without wheezing. Chest X-ray demonstrated bilateral infiltrates. EKG done on admission revealed sinus rhythm (Figure 1).

He was loaded with remdesivir 200 mg IV once, followed by 100 mg IV daily. He was also initiated on ivermectin orally and high-dose methylprednisolone 60 mg IV every 12 hours.

His heart rate remained in the sixties for the first three days and, on day 4 went to a nadir of 33 bpm. Transcutaneous pacer pads were placed on the patient, and atropine at the bedside for severe symptomatic bradycardia. At that time, his oxygen requirements increased to 15 LPM via the non-rebreather mask. The differential diagnosis for the bradycardia was secondary to remdesivir vs ivermectin toxicity, infection-induced bradycardia, or hypoxia-induced bradycardia.

**Figure 2:**
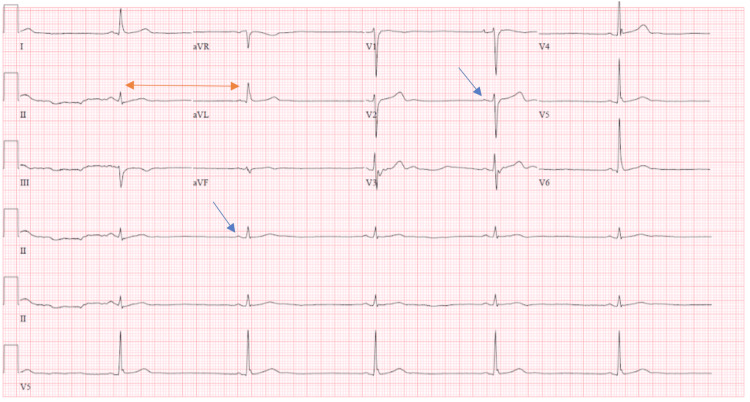
EKG done on day 6 showing severe sinus bradycardia with a heart rate of 31 bpm. Blue arrows showing P waves indicating sinus rhythm; Yellow double arrow pointing out the bradycardia. bpm: beats per minute.

Ivermectin was stopped on day 5, and stable hypoxia persisted. An echocardiogram revealed an ejection fraction of 55-60% with no valvular abnormality or pericardial effusion. Bradycardia persisted with a further nadir of 29 bpm. We extended the course of remdesivir due to the latest update. On day 6, his oxygen requirement increased to 40 L via a high-flow nasal cannula which maintained his oxygenation between 90% and 92%. EKG done at that time revealed persistent severe bradycardia (Figure 2).

**Figure 3:**
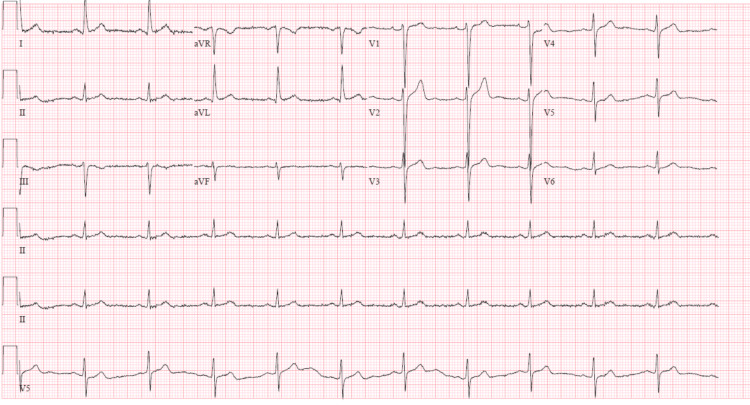
EKG done upon discharge showing normal sinus rhythm with a heart rate of 65 bpm. bpm: beats per minute.

On day 7, the decision was made to discontinue remdesivir and to observe for chronotropic recovery. At that time, the patient still required 40 LPM of oxygen via a high-flow device. On day 10, the patient remained on 40 LPM high flow with SpO2 90-92%. However, his heart rate recovered and was maintained in the high 50s to low 60s, with no further bradycardic episodes noted on telemetry throughout the rest of the hospitalization. Therefore, there was no further requirement for atropine or transcutaneous pacer pads at the bedside. On day 14, the patient's oxygen requirement started to decrease. It continued to reduce gradually throughout the rest of the hospitalization with his heart rate in the high 60s to low 70s prior to discharge. An EKG done prior to discharge demonstrated sinus rhythm (Figure 3).

He was subsequently discharged home on 3 LPM oxygen via nasal cannula with plans to follow up with his primary care provider post quarantining.

## Discussion

Remdesivir is a prodrug of a cyano-adenosine nucleoside analog. It inhibits termination and inhibition of viral activity by inserting itself into SARS-CoV-2 RNA chains. Several clinical trials have shown that treatment of COVID-19 pneumonia using remdesivir therapy is associated with faster recovery compared to placebo. A large number of studies were done that showed the benefits of remdesivir in COVID-19-induced pneumonia. Hence it was extensively used during the pandemic [1]. However, several adverse effects were noted, ranging from cardiovascular to hypersensitivity and anaphylaxis. Notable cardiovascular adverse effects include bradycardia, atrial fibrillation, supraventricular arrhythmias, and other nonspecific arrhythmias [2]. There is limited literature available on remdesivir-induced bradycardia. However, upon extensive literature search, we found two studies [2,3] and four cases [1], [4,5] of COVID-19 pneumonia treated with remdesivir developing bradycardia. In most of these cases, bradycardia resolved within 1-2 days of holding remdesivir, which correlated with the half-life of remdesivir [2]. In 2020, a single-center prospective observational study was done to evaluate the incidence and clinical impact of arrhythmic events in hospitalized patients receiving remdesivir treatment for COVID-19. The study showed that 21% of patients developed sinus bradycardia, with 10% developing atrial fibrillation [2]. According to the study, sinus bradycardia following remdesivir administration does not impact patients' prognosis in terms of ICU admission and in-hospital mortality [2].

Another study compared the cases of bradycardia reported in COVID-19 patients exposed to remdesivir with those COVID-19 patients treated with hydroxychloroquine, lopinavir/ritonavir, tocilizumab, or glucocorticoids. Results showed 94 had bradycardia (31%) out of 2603 patients with remdesivir prescribed in COVID-19 patients. Most of the 94 reports were severe (75, 80%), and in 16 reports (17%), evolution was fatal. Compared with hydroxychloroquine, lopinavir/ritonavir, tocilizumab, or glucocorticoids, the use of remdesivir was associated with an increased risk of reporting bradycardia (reporting odds ratios [ROR]: 1.65; 95% CI: 1.23-2.22) [3].

There are several possible mechanisms for the cardiac adverse effects induced by remdesivir. First, an active metabolite of remdesivir is similar to adenosine triphosphate (ATP), which has been shown to reduce sinus node automaticity through vagal stimulation, which could cause sinus bradycardia. Secondly, as an adenosine analog, remdesivir could affect atrioventricular nodal conduction, which could explain the QRS prolongation seen in some cases. However, further studies to evaluate these mechanisms and their effect specifically on sinoatrial node activity are needed [6].

## Conclusions

Recommendations for the use of remdesivir to treat COVID-19 vary and remain controversial. Prolonged use and high dosages may induce cardiotoxicity manifesting as severe bradycardia, as seen in our case report, in a patient with stable oxygen requirement whose bradycardia resolved post discontinuation of remdesivir. Given that current evidence does not support survival benefits, clinicians should remain mindful of the cardiotoxic adverse effects.
